# SRC-RAC1 signaling drives drug resistance to BRAF inhibition in de-differentiated cutaneous melanomas

**DOI:** 10.1038/s41698-022-00310-7

**Published:** 2022-10-21

**Authors:** Eliot Y. Zhu, Jesse D. Riordan, Marion Vanneste, Michael D. Henry, Christopher S. Stipp, Adam J. Dupuy

**Affiliations:** 1 Department of Anatomy and Cell Biology, The University of Iowa, Iowa City, IA USA; 2 Holden Comprehensive Cancer Center, The University of Iowa, Iowa City, IA USA; 3 Cancer Biology Graduate Program, The University of Iowa, Iowa City, IA USA; 4 The Medical Scientist Training Program, The University of Iowa, Iowa City, IA USA; 5 Department of Molecular Physiology and Biophysics, The University of Iowa, Iowa City, IA USA; 6 Department of Biology, The University of Iowa, Iowa City, IA USA

**Keywords:** Predictive markers, Melanoma

## Abstract

Rare gain-of-function mutations in *RAC1* drive drug resistance to targeted BRAF inhibition in cutaneous melanoma. Here, we show that wildtype RAC1 is a critical driver of growth and drug resistance, but only in a subset of melanomas with elevated markers of de-differentiation. Similarly, SRC inhibition also selectively sensitized de-differentiated melanomas to BRAF inhibition. One possible mechanism may be the suppression of the de-differentiated state, as SRC and RAC1 maintained markers of de-differentiation in human melanoma cells. The functional differences between melanoma subtypes suggest that the clinical management of cutaneous melanoma can be enhanced by the knowledge of differentiation status. To simplify the task of classification, we developed a binary classification strategy based on a small set of ten genes. Using this gene set, we reliably determined the differentiation status previously defined by hundreds of genes. Overall, our study informs strategies that enhance the precision of BRAFi by discovering unique vulnerabilities of the de-differentiated cutaneous melanoma subtype and creating a practical method to resolve differentiation status.

## Introduction

Cutaneous melanoma largely depends on MAPK-signaling, with roughly half of patients harboring the V600E/K activating mutation in BRAF protein. Targeted inhibition of oncogenic *BRAF*^V600^ along with MEK, which is directly downstream of BRAF, is a mainstay of treatment for cutaneous melanoma with mutated BRAF. However, clinical response is not uniform, and most patients progress within two years of treatment^1^. A potential mechanism of drug resistance is through RAC1, a member of the Rho family of small signaling GTPases. RAC1 is a signaling hub and contributes to many biological processes^2^. Hyperactive RAC1, for example, *RAC1*^P29S^, is a previously-described driver of drug resistance to BRAF inhibition (BRAFi)^3–6^. However, this mutation is rare—present in around 5% of samples in the 448 TCGA skin cutaneous melanoma samples^7,8^. Nonetheless, heterogeneity in the degree of RAC1 signaling may explain the variation in response to BRAFi. For instance, we have previously demonstrated that amplifying RAC1 signaling through overexpression of its GEF, *VAV1*, drives drug resistance to BRAFi in cutaneous melanoma. We have also shown that *RAC1* expression can be used to predict response to BRAFi^9–11^.

The mechanism by which RAC1 drives resistance to BRAFi is not fully understood. PAK1, RAC1’s canonical downstream target, has been described to drive drug resistance^12^. Alternatively, RAC1 drives the formation of dendritic actin, which leads to decreased dependence on MAPK in cutaneous melanoma^6^. RAC1’s ability to regulate actin is consistent with RAC1’s ability to elicit a mesenchymal switch in cutaneous melanoma through the recruitment of the SRF/MRTF transcription factor, whose activity is regulated by actin dynamics^13^. SRF/MRTF cooperate with other master regulators of the mesenchymal program and have been implicated in drug resistance to BRAFi^14^. While melanoma cells are neither epithelial nor mesenchymal, they can be classified based on the expression programs that typify these states^15,16^. Mesenchymal melanomas, driven by various mesenchymal-related transcriptional factors, such as Zeb1, TGF-β, AP-1, and Yap/Taz, have been shown to be more resistant to BRAFi^13,17–21^. These observations led us to speculate that wildtype *RAC1* signaling can drive innate resistance to BRAFi.

We found that RAC1 is a driver of growth and innate drug resistance to BRAFi in some melanoma cell lines and that the reliance on RAC1 was associated with the de-differentiated phenotypic state. De-differentiation was also predictive of response to the co-inhibition of SRC and BRAF. We shed light on this connection by showing that RAC1 and SRC critically maintain melanoma de-differentiation. While our study is focused on drug resistance to BRAFi, our findings could also inform strategies that overcome drug resistance to immune checkpoint inhibition (ICI). The association between de-differentiation and ICI-resistance has been emphasized in several studies^22,23^. Given that SRC and RAC1 maintain de-differentiation, it remains to be seen whether targeting these pathways could also influence sensitivity to ICI.

The unique vulnerabilities of the de-differentiated subtype may inform strategies that sensitize these melanomas to BRAFi or ICI. Unfortunately, prior reports identify de-differentiated melanomas using large gene sets derived from transcriptome analysis^15,16,24,25^. This approach is not readily translated to a clinical setting where pathological subclassification of tumors typically relies on a small number of markers. To address this limitation, we construct a binary classification using a small set of genes based on melanocyte differentiation. We evaluate the accuracy and clinical relevancy of our classification strategy using cell line and patient datasets.

## Results

### RAC1 drives growth of melanoma cells in standard conditions or during BRAF inhibition

To test the importance of RAC1 for drug resistance, we knocked down RAC1 in a panel of nine *BRAF*^V600E^ cutaneous melanoma cell lines and assessed their growth in the presence or absence of a targeted inhibitor of *BRAF*^V600E^, vemurafenib (VEM). Most of the cell lines we studied belonged to the NCI-60 panel. Three cell lines, PDX10, vRPP1, and vRPP3 were generated in-house (see “Methods”). Importantly vRPP1 and vRPP3 are VEM-resistant derivatives of A375. vRPP3 served as a positive control as it harbors a heterozygous N92I mutation in *RAC1*, confirmed via Sanger sequencing (Supplementary Fig. 1a). N92I is a known gain-of-function mutation for *RAC1*^4,26^. We confirmed that *RAC1*^N92I^ drives drug resistance to VEM as does *RAC1*^P29S^ (Supplementary Fig. 1b). vRPP1 has wildtype *RAC1*. Another important consideration is that neither vRPP1 nor vRPP3 harbor *NRAS* mutations or *BRAF* genomic alterations, which drive drug resistance through MAPK-reactivation^9,27^. Lastly, the panel of cell lines we profiled differed in their sensitivity to BRAFi. RPMI7951, A2058, vRPP1, vRPP3 were among the most drug-resistant and proliferated even in >1 μM of VEM (Supplementary Fig. 2).

**Fig. 1:**
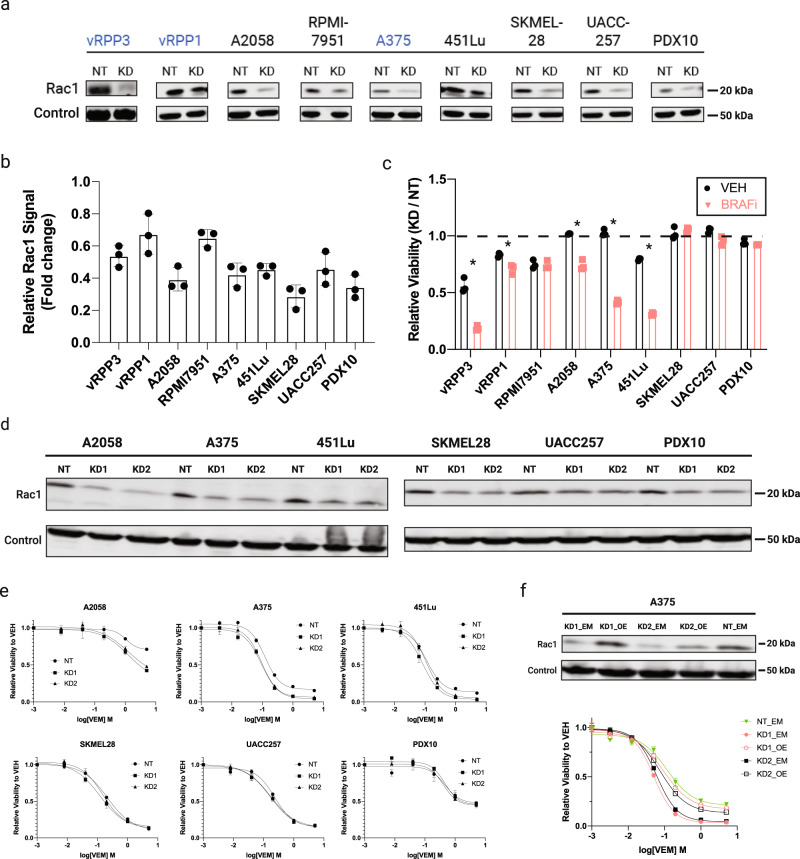
RAC1 signaling is a non-uniform driver of growth and drug resistance to BRAFi. **a** Western blot of RAC1 in cell lines transduced with RAC1-shRNA or non-targeting control. Color emphasizes that vRPP1 and vRPP3 are sublines of A375 (NT non-targeting, KD knockdown). **b** Fold change of RAC1 protein levels in KD compared to NT. **c** Viability of cell lines with *RAC1*-knockdown relative to NT-control in VEM or vehicle (VEH) after 34–72h. The timepoints shown correspond to the maximum signal intensity recorded over 72h (see Supplementary Fig. 2 for experimental details). **d** Confirmation of *RAC1*-knockdown via western blot for the indicated cell lines four days after transduction of viral shRNA vector (NT non-targeting, KD1/2 *RAC1*-targeting shRNA 1 or 2). **e** 72h dose-response curves for cells transduced with either NT, KD1, or KD2 shRNAs. Cells were seeded 48h post-transduction and either VEH or drug were added 24h later. Viability is normalized to VEH. **f** (Top) Western blot of RAC1 in different modified versions of A375 (NT non-targeting shRNA, EM empty vector, KD1/2 *RAC1*-targeting shRNAs, OE overexpression of shRNA-resistant *RAC1*). (Bottom) 72h dose-response curves for variants of A375 described above. Error bars in this figure denote the standard deviation. Asterisk denotes unpaired two-tailed Student’s *t* test adjusted *p* value of <0.05.

Knockdown of *RAC1* was confirmed via western blot. We achieved variable knockdown in the panel of cell lines (Fig. 1a, b). However, the growth of the intrinsically drug-resistant melanomas (A2058, RPMI7951, vRPP1, and vRPP3) was reduced by *RAC1-*knockdown either in standard conditions (RPMI7951, vRPP1, vRPP3) and/or during BRAFi (A2058, vRPP3). Among the BRAFi-sensitive cell lines, only two out of the five cell lines (A375 and 451Lu) were further sensitized to VEM by *RAC1*-knockdown (Fig. 1c and Supplementary Figs. 2 and 3).

To determine if this result was reproducible, we repeated the knockdown of RAC1 with the same shRNA and another *RAC1*-targeting shRNA and determined the impact of *RAC1*-knockdown using a dose-response assay. We performed this experiment on six cell lines used previously (A2058, 451Lu, A375, SKMEL28, UACC257, and PDX10). Again, we achieved variable *RAC1-*knockdown across the panel of cell lines (Fig. 1d). Nonetheless, the outcome of the dose-response experiments was consistent with our prior observation that *RAC1*-knockdown sensitized a subset of cutaneous melanomas to BRAFi (Fig. 1c, e). Finally, we expressed an shRNA-resistant form of *RAC1* in A375 expressing either *RAC1-*KD1 or *RAC1-*KD2 shRNAs. The shRNA-resistant cDNA rescued RAC1 protein levels and increased drug resistance to BRAFi (Fig. 1f).

**Fig. 2:**
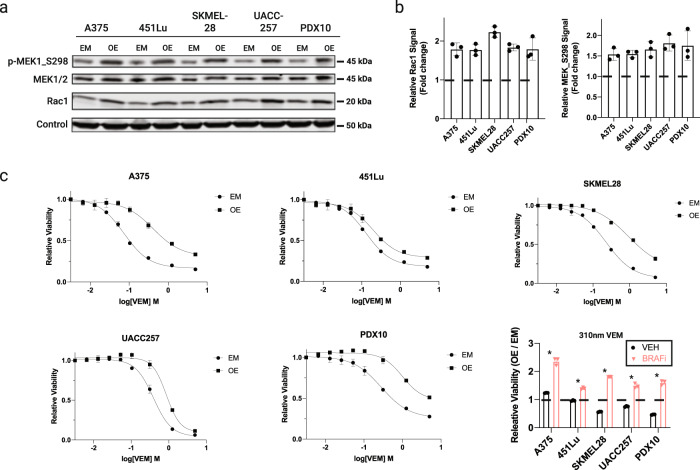
*RAC1*^P29S^ affects the growth of cutaneous melanoma cells in standard conditions or during BRAFi. **a** (Left) Western blot of RAC1, MEK1/2, and p-MEK1_s298 in cell lines stably transfected with empty vector or *RAC1*^P29S^ expression vector (EM empty vector, OE overexpression of *RAC1*^P29S^). **b** Fold change of RAC1 (left) and MEK_S298 (right) protein levels in OE compared to EM-modified cells. **c** Dose-response curves of cell lines with enforced expression of *RAC1*^P29S^ or empty-vector control in VEM over 3–5 days. Viability is normalized to vehicle-treated cells. (Bottom right) Viabilities of the dose-response curves shown in **c** at a fixed concentration of 310 nm VEM. Error bars in this figure denote the standard deviation. Asterisk denotes unpaired two-tailed Student’s *t* test adjusted *p* value of <0.05.

We were curious why some of the melanoma cell lines we studied were not sensitized to BRAFi upon *RAC1*-knockdown. Hyperactive RAC1-signaling is a well-established driver of drug resistance, but we wondered if this mechanism works in the cell lines where *RAC1*-knockdown had minimal impact on cell proliferation. To test this, we enforced expression of hyperactive RAC1, *RAC1*^P29S^, in the three melanoma cell lines (SKMEL28, UACC257, PDX10) where *RAC1*-knockdown had little impact. As positive controls, we also expressed *RAC1*^P29S^ in two BRAFi-sensitive melanomas (A375 and 451Lu) in which *RAC1*-knockdown did impact sensitivity to BRAFi. We confirmed enforced expression via western blot (Fig. 2a, b). To determine whether the modified cell lines have increased RAC1-signaling, we measured levels of p-MEK_S298 in parental empty vector and modified cell lines. The S298 site on MEK is a well-established target of PAK1, which is a direct target of RAC1^28,29^. Indeed, this marker of PAK1 activity was elevated in cell lines with enforced expression of *RAC1*^P29S^ relative to that of empty vector (Fig. 2a, b). We found that *RAC1*^P29S^ could drive BRAFi resistance in all five melanoma cell lines (Fig. 2c). However, *RAC1*^P29S^ unexpectedly slowed the growth of three melanomas that were not impacted by RAC1-depletion (Fig. 2c). *RAC1*^P29S^ also greatly altered the morphology of these three melanoma lines (Supplementary Fig. 4).

Overall, these data highlight the importance of RAC1 in driving growth or innate drug resistance to BRAFi in cutaneous melanoma cells. Moreover, our findings suggests that melanomas differ with respect to utilization of RAC1-signaling.

### Reliance on RAC1 is linked to melanoma differentiation

**Fig. 3:**
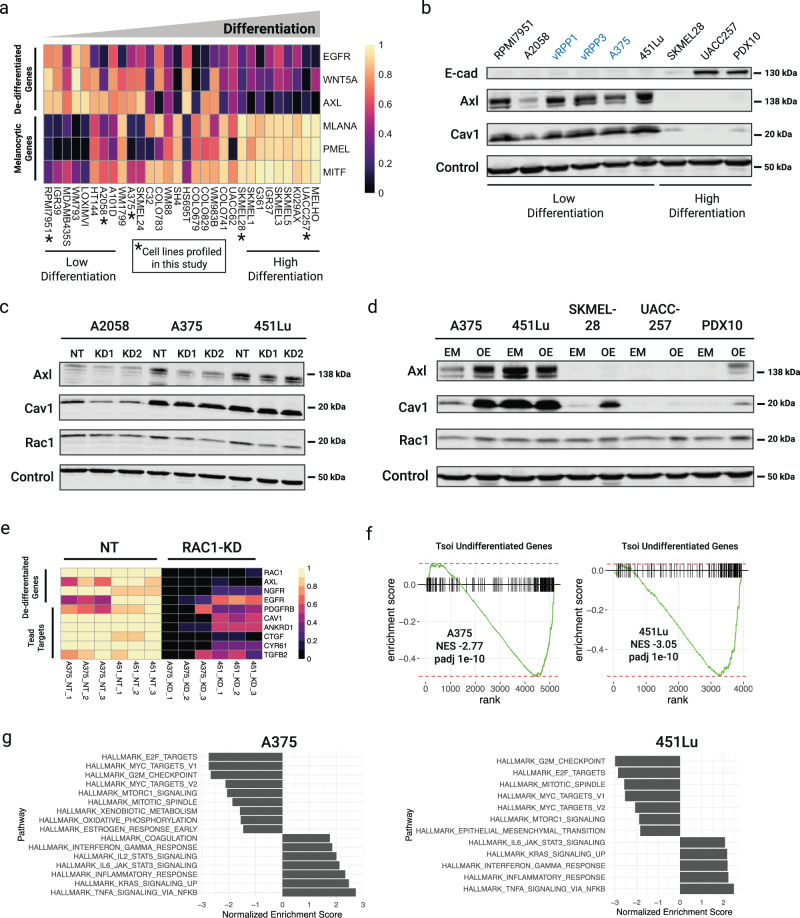
Melanoma differentiation state correlates with RAC1 dependence and is modulated by RAC1 signaling. **a** Min-max normalized expression of select differentiation-associated genes across *BRAF*^V600^ melanomas. **b** Western blot of proteins reflecting differentiation status across melanoma cell lines profiled in this study. **c** Western blot of melanoma differentiation markers in de-differentiated melanoma cell lines 72h post-transduction with either a non-targeting control or different shRNAs targeting RAC1, denoted by NT, KD1, or KD2, respectively. **d** Western blot of melanoma differentiation markers in five melanoma cell lines with enforced expression of either empty vector or *RAC1*^P29S^ (EM empty vector, OE overexpression of *RAC1*^P29S^). **e** Min-max normalized expression of select de-differentiation genes altered by knockdown of *RAC1*. Data represent three biological replicates of cells transduced with NT or KD1. **f** Enrichment plot of Tsoi undifferentiated melanoma gene set in genes differentially expressed upon *RAC1-*knockdown. **g** Hallmark gene sets enriched in genes differentially expressed upon *RAC1-*knockdown. Only pathways with adjusted *p*-value of <0.05 are shown.

Several studies have identified gene expression signatures correlated with clinical outcomes in cutaneous melanoma patients. Notably, the de-differentiated melanoma subtype typified by a MITF^lo^/AXL^hi^ or MITF^lo^/NGFR^hi^ transcriptional state is well-described to be more resistant to BRAFi^16,30–36^. To determine whether these subtypes relate to RAC1-dependence, we queried CCLE gene expression data for markers of melanocyte differentiation and de-differentiation in the cell lines we studied (Fig. 3a). AXL, EGFR, and WNT5A are markers and/or drivers that are elevated in inherently drug-resistant cutaneous melanomas^19,35,37–39^. Indeed, the cell lines affected by *RAC1*-knockdown showed elevated expression of de-differentiated genes and decreased expression of melanocyte differentiation genes.

To find other markers of the de-differentiated state, we mined RPPA protein array data and identified proteins that separated de-differentiated from differentiated melanomas. We found that Cav1 and E-cadherin (*CDH1*) were upregulated in de-differentiated or differentiated melanomas, respectively (Supplementary Fig. 5a, b). E-cadherin is under the direct control of MITF, while *CAV1* is a downstream target of the TEAD family of transcription factors^40,41^. Next, we estimated the melanoma differentiation states of the cell lines we studied by western blot. We found that the cell lines that depended on RAC1 in the presence and/or absence of BRAFi had elevated Cav1 and AXL and no E-cadherin (Fig. 3b).

Lastly, we wondered whether intrinsic or enforced RAC1 signaling regulates melanoma differentiation. We found that knockdown of *RAC1* decreased AXL and/or Cav1 (Fig. 3c). Conversely, overexpression of *RAC1*^P29S^ increased these genes in both differentiated and de-differentiated melanomas (Fig. 3d). The latter observation has been previously demonstrated in mouse melanocytes^13^. To further interrogate the role of RAC1 in maintaining de-differentiation, we performed RNAseq on A375 and 451Lu with shRNA-depleted RAC1 and respective controls (Supplementary Data). Interestingly, depleting RAC1 resulted in the downregulation of several previously reported markers of de-differentiation (Fig. 3e). Globally, genes differentially expressed upon *RAC1-*knockdown negatively enriched for the Undifferentiated melanoma gene set defined by Tsoi et al. (Fig. 3f). *RAC1*-knockdown also influenced other pathways as demonstrated by enrichment of GSEA Hallmark gene sets shown in Fig. 3g.

Overall, these findings suggest that even without gain-of-function mutations. RAC1 helps de-differentiated melanomas grow and/or withstand BRAFi and helps maintain the de-differentiated state.

### RAC1-dependence is correlated with distinct pharmacologic vulnerabilities

**Fig. 4:**
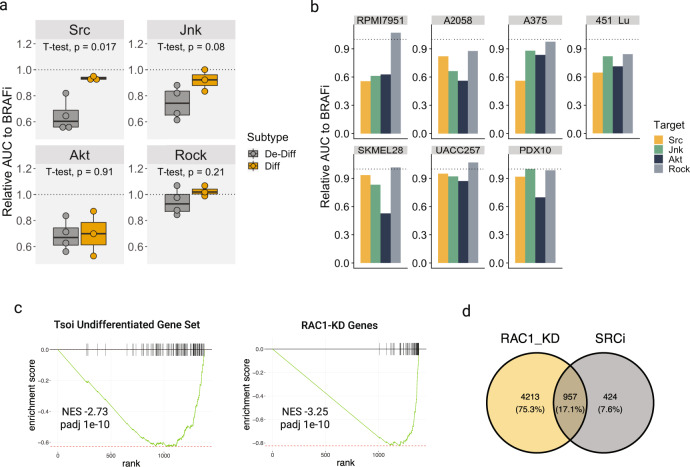
Inhibiting de-differentiation increases the efficacy of BRAFi. **a** Each point is the relative area under the curve (AUC) of the BRAFi dose-response curve in combination with drugs targeting the proteins indicated in the panel headers for a given cell line. The dotted line indicates no difference compared to BRAFi alone. Colors indicate whether a cell line belongs to the de-differentiated or differentiated class. Eight concentrations of VEM, up to 5 uM, were used to generate the dose-response curves. For the drug combinations, a constant dose of 1 uM Saracatinib, 1 uM JNK-IN-8, 1 uM of MK2206, or 4 uM of Fasudil was added to varying concentrations of VEM. The edges of the boxes in this plot denote the 1st and 3rd quartiles, and the line indicates the 2nd quartile. **b** Same dataset visualized in **a** shown for each cell line. The *y*-axis, which presents the area under the curve, represents the entire area under the dose-response curve shown in Supplementary Fig. 6. The lower the area, the more sensitive a cell line is to the respective drug. As detailed in the methods, each dose was measured in triplicates. The area is one value, so no statistics can be performed. **c** Enrichment plots of RAC1-responsive gene set and Tsoi undifferentiated gene set in genes differentially expressed upon SRCi. **d** Venn diagram of the number of differentially expressed genes shared or not upon *RAC1-*knockdown or SRCi.

Our results imply that de-differentiated melanomas would be vulnerable to compounds that inhibit RAC1. Unfortunately, RAC1 is a small signaling GTPase and cannot be targeted directly due to the lack of specificity and/or potency of proposed strategies^42^. Instead, we sought to indirectly inhibit RAC1 signaling by blocking upstream or downstream components of RAC1 signaling. We narrowed our focus on drugs that have been previously reported to sensitize melanomas to BRAFi^9,10,17,20,32,43–46^. These included saracatinib, JNK-IN-8, and MK-2206, which are selective inhibitors of SRC kinases, Akt, and JNK, respectively. We also included Fasudil, a ROCK inhibitor, since Rho signaling has also been linked to BRAFi resistance^14^. We treated four de-differentiated and three differentiated melanomas with VEM alone or in combination with the inhibitors mentioned above. We found that only de-differentiated cell lines were sensitized to BRAFi by SRC inhibition (SRCi), while the differentiated melanomas only showed a modest decrease in the AUC compared to that of VEM alone (Fig. 4a, b and Supplementary Fig. 6).

To inform the mechanism by which SRCi sensitizes melanoma cells to BRAFi, we performed RNAseq on A375 treated with 1 uM saracatinib (Supplementary Data). The genes differentially expressed upon SRCi negatively enriched for the Tsoi undifferentiated gene set. These genes were also negatively enriched for a set of genes downregulated by *RAC1-*knockdown in both A375 and 451Lu. We call this collection of genes the RAC1*-*responsive gene set (Fig. 4c and Supplementary Data). Only genes with absolute log2FC of < −2 were used to generate this gene set. Moreover, around 60% of genes that are significantly differentially expressed with SRCi are shared by *RAC1-*knockdown (Fig. 4d).

These results suggest that SRCi can partially suppress the output of RAC1, reduce de-differentiation, and increase sensitivity to BRAFi in cutaneous melanoma.

### A practical approach to resolving differentiation status in cutaneous melanoma

Given the selective sensitivity to SRCi, divergence in RAC1 utilization, and association with drug resistance in de-differentiated melanomas, we aimed to create a practical strategy to determine melanoma differentiation status. Melanoma differentiation subtypes proposed by Hoek, Veraillie, or Tsoi use hundreds of genes, which is not feasible for a clinical test. We sought to find a small set of genes, with comparable performance to the larger gene sets, that is suitable for a cheap and practical clinical test. We intentionally excluded MITF from our gene set because MITF is a transcription factor and its transcriptional competency and stability are regulated by multiple mechanisms^47^. Instead, we reasoned that MITF target genes may serve as more specific indicators of melanocyte differentiation.

The basis for our small gene set is a study performed by Veraillie et al., which revealed that TEADs and AP-1 transcription factors maintains melanoma de-differentiation and the MITF and SOX10 transcription factors maintains melanoma differentiation^24^. Interestingly, melanomas dependent on TEAD1 tended to also depend on RAC1 according to Depmap CRISPR dependency scores (Supplementary Fig. 7).

**Fig. 5:**
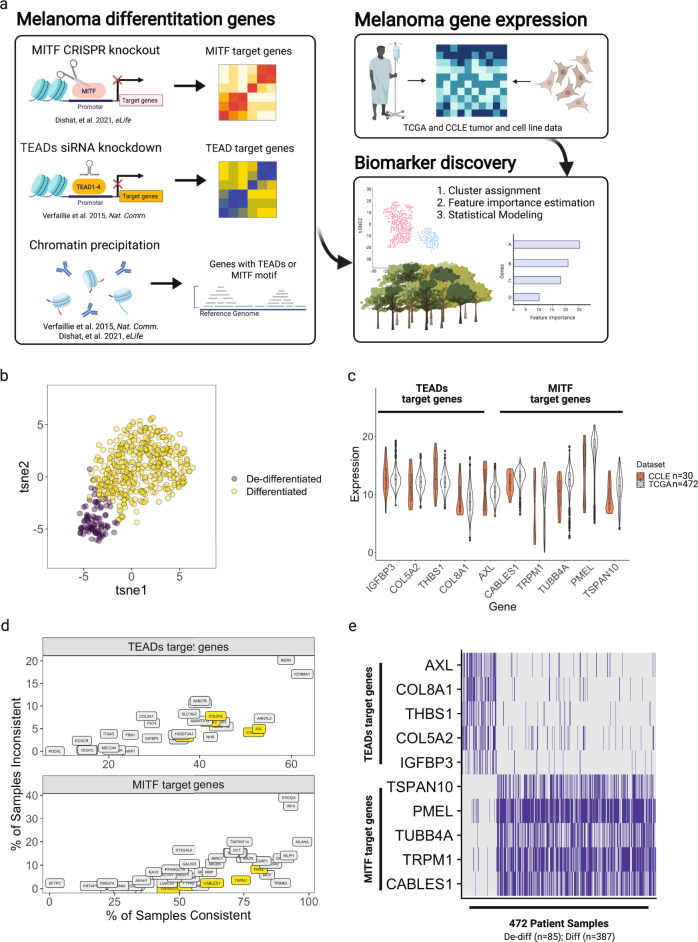
Identification of a small gene set to approximate melanoma differentiation state. **a** Data mining strategy. Created using BioRender. **b** t-SNE of TCGA melanoma samples using ~400 invasive and proliferative genes. Cluster assignment determined by K-means. **c** Comparison of expression of small gene set between CCLE cell lines and TCGA patient data. The edges of the boxes in this plot denote the 1st and 3rd quartiles, and the line denotes the 2nd quartile. **d** Percentage of samples that candidate genes were positive for the class it defines. **e** Binary expression of the small gene set in TCGA samples. Genes were set to one if the normalized counts belonged to the upper tertile for each gene across the TCGA dataset and zero otherwise.

Existing gene signatures used to define melanoma differentiation rely on the expression of hundreds of genes^15,16,25,48^. Instead, we sought to use genes that are confirmed TEAD and MITF/SOX10 targets to reduce the risk of overfitting (Fig. 5a). This strategy differs from that of melanoma clinical diagnostic scoring systems, such as DecisionDx, which utilize genes that best explain relevant metrics, such as progression-free survival^49^.

Using TCGA SKCM RNAseq data, we separated tumors into two classes based on a reduced set of de-differentiation/differentiation genes that cluster tightly among themselves. We took this approach to achieve the cleanest separation of the two subtypes. A t-SNE visualization using this set of ~400 de-differentiation/differentiation genes showed the separation of these two states (Fig. 5b).

Next, we sought to reduce the number of genes used in the classifier by selecting TEADs- or MITF- regulated genes that, on their own, could separate the two classes using a random-forest based analysis. We also selected genes with expression values comparable to that of cutaneous melanoma cell lines in the CCLE dataset to prevent selecting genes that are mostly expressed by stromal cells (Fig. 5c). To simplify the classification, we converted the expression of each gene to a binary value where samples within the top tertile of expression for a given gene was set to one, and zero otherwise. We settled on five genes representative of the de-differentiated and differentiated class that showed high specificity (Fig. 5d). A visualization of the binary version of the TCGA SKCM gene expression dataset using just the ten genes we have identified is shown in Fig. 5e.

**Fig. 6:**
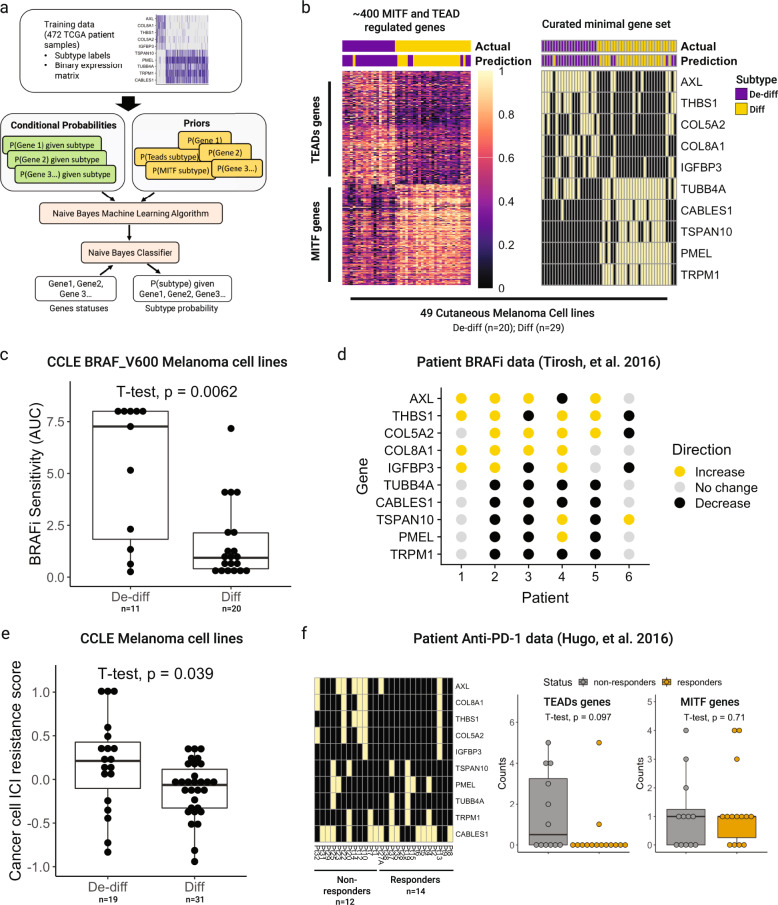
Differentiation-based Bayesian classification approach for cutaneous melanoma. **a** Bayesian learning strategy to generate a binary classification algorithm for cutaneous melanoma. **b** (Left) Heatmap of invasive and proliferative genes used for clustering assignment in CCLE cutaneous melanoma cell lines. Top annotation bar denotes subtype assignment determined by expanded gene set, and bottom annotation bar denotes subtype assignment from the Bayesian classifier using curated gene set. (Right) Same heatmap but with the binary expression of the curated gene set. **c** AUCs for Vemurafenib across melanomas belonging to de-differentiated or differentiated class obtained from CCLE. **d** Direction of change for the small gene set in paired pre-treatment and progression patient samples on vemurafenib. An increase or decrease is defined as a greater than 50% change in normalized gene expression values. **e** Cancer cell ICI-resistance score as reported by Jerby-Arnon et al. across de-differentiated and differentiated melanomas. **f** (Left) Binary expression of our small gene set in patients who responded or progressed on anti-PD1 therapy from Hugo et al. dataset. Expression for a given gene was set to one if it belonged to the 25th percentile. (Right) Total counts of TEADs or MITF genes in non-responders vs. responders. Alternative visualization of heatmap shown on the left. For the box plots in this figure, the edges of the boxes denote the 1st and 3rd quartiles, and the line denotes indicates the 2nd quartile.

We then fed these genes into the Naïve Bayes machine learning algorithm to build a model that could classify a melanoma based on the binary expression of our ten genes. Intuitively, the algorithm generates a statistical model that computes the probability of being in each class given the expression for a set of genes based on the associations between each gene and class (Fig. 6a). Our training data was the TCGA SKCM gene expression dataset, and the test data was the CCLE cutaneous melanoma gene expression dataset. Our model achieved a balanced accuracy of 87% (Fig. 6b).

Finally, to highlight the clinical value a binary differentiation-based classification system, we profiled drug response to BRAFi in de-differentiated vs. differentiated melanomas. We found that de-differentiated melanomas tended to be the most innately drug-resistant (Fig. 6c). Furthermore, in 5/6 patients of a previously published dataset, de-differentiated genes increased in cancers that progressed on BRAFi compared to pre-treatment (Fig. 6d)^34^.

With respect to ICI, one study derived a cancer cell ICI drug resistance program using large-scale scRNAseq data and computed the enrichment of this signature in CCLE melanoma cell lines^50^. When we compared these scores across the two subtypes, we again found that the de-differentiated subtype tended to be more drug-resistant (Fig. 6e). Previous gene expression profiling of patient tumors that responded to ICI vs. those that progressed found that AXL and E-cadherin correlated or anti-correlated with ICI-resistance, respectively^51^. However, our curated gene set could not separate responsive from progressive disease using the same dataset (Fig. 6f).

## Discussion

The de-differentiated subtype of cutaneous melanoma is a recurrent transcriptional state linked to drug resistance to BRAFi. Several studies have shown that melanomas belonging to the de-differentiated state have increased expression of many markers that either drive or associate with resistance to BRAFi. Notable examples include, AXL, NGFR, EGFR, PDGFRB, WNT5A, ZEB1, SOX9^14,18,32,35,37–39,52^. This subtype was originally described by Hoek et al. as the invasive subtype within their invasive/proliferative classification system^15^. Veraillie et al. elucidated that the AP-1/TEAD served as master regulators of the de-differentiated state.

Here, we show that RAC1 tends to be more important in de-differentiated melanomas for growth in standard conditions and/or during BRAFi. This pattern of dependence may owe to wildtype RAC1’s ability to maintain the de-differentiated state. This knowledge is important as targeting the RAC1-pathway may sensitize an intrinsically therapy-resistant subtype of melanoma to BRAFi. We also observed that RAC1 signaling opposes the proliferative effect of MITF because *RAC1*^P29S^ suppressed the growth of differentiated melanomas. This result is consistent with a past study that used a marine-organism-derived compound, Plitidepsin, to hyperactivate RAC1 signaling in the differentiated cell lines, SKMEL28 and UACC257^53^. Perhaps a negative feedback loop exists between RAC1 and MITF, as the growth of differentiated melanomas critically depends on MITF and that deletion of MITF results in the rampant activation of Rho family GTPases^40^.

Our evaluation of different BRAFi-based drug combinations suggests that inhibiting SRC kinases with saracatinib can sensitize, de-differentiated melanomas to BRAFi. Although saracatinib is not FDA approved, other inhibitors of SRC kinases such as dasatinib may have similar clinical impact. Indeed, we found that dasatinib increased the effect of BRAFi (Supplementary Fig. 8). However, dasatinib inhibits many kinases beyond the SRC family^54^. Thus, it is unclear how dasatinib inhibits proliferation. Previous studies have demonstrated the promise of co-inhibiting SRC kinases and BRAF both in vitro and in vivo^9,10,32,43,55^. SRCi may work through inhibiting the transmission of extracellular matrix (ECM) stiffness, activation of Hippo kinases, or suppression of RAC1 signaling through regulation of RAC1-specific GEFs, RhoGDIs, or CUL3^9,10,55–58^. Here, we show that SRCi suppresses de-differentiation (Fig. 4c, d).

Our findings are clinically meaningful because ECM remodeling and YAP transcriptional signatures are elevated in patient tumors that have progressed on VEM^30^. Upregulation of these processes has been described as the most recurrent features of MAPK-redundant drug resistance^31^. In a greater context, TEADs’ ability to promote resistance to MAPK-targeting therapies seems to conserved across cancer types^59,60^. Strategies to target TEADs are currently limited, but inhibiting SRC kinases appears to be the most promising, as suggested by a recent study that performed pharmacogenomic analysis on Yap-On vs. Yap-Off tumors^61^. Nonetheless, there are inhibitors of TEADs under development^62^. An alternative strategy is to target the epigenetic regulators of melanoma differentiation^63^.

A limitation of our experiments is that we only examined the short-term benefit of SRCi in combination with the BRAFi. It is known that differentiated melanomas undergo drug-induced de-differentiation, and TEADs mediate drug resistance in melanomas that have undergone de-differentiation^21,35^. Thus, it is unclear what impact SRCi will have on phenotypic plasticity during the emergence of drug resistance.

Since melanoma differentiation influences drug resistance, knowing how to classify a patient’s cancer would have high clinical value. To simplify this task, we have identified a small set of genes based on the master regulators of cutaneous melanoma transcriptional states, i.e., TEADs and MITF. Certainly, a binary classification system is a simplification of multiple subtypes^64^. Moreover, at the single-cell level, melanoma tumors are composed of a mixture of subtypes, while we are proposing to define subtypes based on the population average^34,36,65^. However, our data would suggest that a bulk estimate can still have clinical value as melanomas classified, as de-differentiated are resistant to multiple therapies and have distinct signaling vulnerabilities (Fig. 6c–f).

Several studies have underscored the connection between ICI and de-differentiation: Indirectly, exposure to inflammatory cytokines or cytotoxic T-cells induced de-differentiation in melanoma cells^16,38,66^. Directly, a NGFR transcriptomic signature is elevated in persister cells that survive ICI treatment and that among four mouse melanomas that mimic human transcriptomic profiles, de-differentiated cancers were resistant to anti-PD1 therapy^23,67^. Clinical samples also support the connection between de-differentiated cancers and ICI-resistance, as a recent study on 94 patient tumors at baseline and on ICI treatment revealed that de-differentiation was the only transcriptomic signature that was associated with MHC class I downregulation, which they define as a hallmark of resistance to anti-PD1 therapy^22^. Finally, at the fundamental level, hyperactive RAC1 has been shown to increase PD-L1 protein levels^68^.

In summary, our work highlights the SRC-RAC1 axis as a vulnerability in de-differentiated melanomas. Additionally, we offer a practical solution to resolve melanoma differentiation status. Despite extensive data on the behavior and molecular features of cutaneous melanoma subtypes, this knowledge is still not utilized in the clinic. Our work seeks to bridge the gap through biomarker discovery and the characterization of the unique vulnerabilities of the de-differentiated subtype.

## Methods

### Cell lines

A375, 451Lu, and SKMEL28 were obtained from ATCC. UACC257, RPMI7951, and A2058 were obtained from NCI cell line repository. PDX10 was obtained from a patient-derived xenograft from a patient with *BRAF*^V600E^, *NRAS* WT, cutaneous melanoma. PDX10 was confirmed to be a human cell line via STR analysis. vRPP1 and vRPP3 are drug-resistant sub-lines of A375. We generated vRPP1 and vRPP3 by isolating colonies that formed while parental A375 was treated with a cytostatic dose of VEM. We have confirmed via sanger sequencing that vRPP1 and vRPP3 are *BRAF*^V600E^ and *NRAS* WT. vRPP3 harbors *RAC1*^N92I^ and vRPP1 is *RAC1* WT. A375, 451Lu, A2058, and RPMI7951 were cultured in Gibco DMEM, supplemented with penicillin/streptomycin, and 10% FBS. SKMEL28 was cultured in Gibco DMEM, supplemented with penicillin/streptomycin, 10% FBS, Sodium pyruvate, and non-essential amino acids. PDX10 and UACC257 were cultured in Gibco RPMI, supplemented with penicillin/streptomycin, and 10% FBS. Written informed consent was obtained from the patient to create PDX10 cell line for research use. We complied with all relevant ethical regulations in creating this cell line. PDX10 was obtained through the University of Iowa Holden Comprehensive Cancer Center’s Melanoma: Skin and Ocular Tissue Repository (MAST), an Institutional Review Board-approved biospecimen repository and data registry (IRB protocols 201708847 and 200804792).

### RAC1-knockdown real-time viability assay

Knockdown of *RAC1* was performed with lentivirus containing *RAC1*-shRNA (KD1) or non-targeting shRNA (NT) in a 6-well format. 48h post-transduction, cells were seeded in a 96-well plate. 24h after seeding, cells were treated with either DMSO or indicated dose of VEM and monitored for 72h after treatment. Viability was assessed with RealTime-Glo, a luminescence-based reagent. Different doses of VEM were used for each cell line based on their intrinsic drug sensitivities. Different doses were used to better assess the impact of *RAC1*-knockdown. Using too high a dose of VEM would mask the impact of *RAC1-*knockdown on VEM response. The luminescence signal for different cell lines became saturated at different times, leading to different end time points for graphs shown in Supplementary Fig. 2. No antibiotic selection was performed as some cell lines could not survive RAC1-depletion and selection process. Cell lysates were collected 72h post-transduction. Transduction efficiency was confirmed using flow cytometry. The sequence for *RAC1-*KD1 was GATCCGAAGGAGATTGGTGCTGTAAAATTCAAGAGATTTTACAGCACCAATCTCCTTTTTTTTCTAGACAATT. The sequence for *RAC1-*KD2 was GATCCGCAAGAAGATTATGACAGATTATTCAAGAGATAATCTGTCATAATCTTCTTGTTTTTTCTAGACAATT.

### RAC1-knockdown rescue

A375 was first transduced with lentivirus containing KD1 or NT shRNA. 48h later, the cells underwent puromycin selection (1 ug/ml) for 72h. These cells were then transfected with empty vector or shRNA-resistant *RAC1* plasmid using the Qiagen Effectene transfection reagent. Afterwards, cells underwent one week of neomycin selection (500 ug/ml), 48h after transfection. The antibiotic media was changed every 48h. The seed sequences for *RAC1-* KD1 and KD2 shRNAs were AAGGAGATTGGTGCTGTAAAA and CAAGAAGATTATGACAGATTA, respectively. In the shRNA-resistant *RAC1* construct, these sequences were changed to AAAGAAATCGGAGCGGTCAAG and CAGGAGGACTACGATAGGTTA.

### RAC1^P29S^ overexpression

Enforced expression *RAC1*^*P29S*^ was performed using a *piggyBac* transposon-transposase system^69^. Namely, cells were seeded in a 6-well format. 24h later, empty vector or *RAC1*^*P29S*^ plasmid were mixed at 1:5 ratio with the *PiggyBac* transposase plasmid and delivered into cells with either the Qiagen Effectene or Jetoptimus DNA transfection reagent using the standard workflow. Media was changed 6–24h later. Cells were selected with puromycin for six days with media changes every two days.

### Cell viability assay

Drug dose-response curves were generated using the Resazurin reagent. Viability at each dose was measured in triplicate. Cells were seeded in a 96-well plate. Cells were treated with drug 24h later. Data shown represents fluorescent signal detected at day 3–5 normalized to the vehicle-treated wells. For drug combination experiments, all the drug combinations were tested at the same time as BRAFi alone. The assay was performed by putting 100 ul of media and 20 ul of 6x stock (0.15 mg/ml) of resazurin onto cells, followed by a 2h incubation at 37 degrees in a tissue culture incubator.

### Immunoblotting

Whole-cell lysates were separated on Tris-Glycine 4–20% gradient gels (Thermo Fisher) and transferred to nitrocellulose membranes overnight. Blots were blocked in Odyssey Blocking Buffer PBS (Licor) for 1h and incubated with primary antibodies overnight at 4 degrees, followed by 1h incubation at room temperature with secondary antibodies. Blots were imaged using the Odyssey 9210 (Licor). The control antibody was alpha-tubulin (12G10 UIOWA hybridoma bank) for all the blots except the vRPP3 blot in Fig. 1a, which we used beta-actin as the control (Sigma A1978). The other antibodies used were RAC1 (BD 610651), Cav1 (CST 3267S), AXL (CST C89E7), and E-cadherin (RD MAB1838), MEK (CST 9122), and p-MEK_S298 (CST 9128). All antibodies were used at 1:1000 dilution, except for E-cadherin (RD MAB1838), which was used at 1:250 dilution. Western blots were quantified using ImageStudioLite software. All western blots were done in triplicates, derived from same experiments, and processed in parallel. Unprocessed and uncropped blot scans can be found in the Supplementary Information (Supplementary Fig. 9).

### RAC1-knockdown and SRCi differential gene expression analysis

For *RAC1*-knockdown, parental cell lines were transduced with shRAC1 (KD1) or shNT containing lentivirus on day zero. The media was changed on day two. Puromycin antibiotic selection (1 ug/ml) was then performed for three days. Cells were then grown in standard media for three days. RNA was extracted from the cells on day eight. For the SRCi RNAseq experiment, cells were treated with vehicle or 1uM of saracatinib for three days. The Monarch Total RNA Miniprep kit was used to extract the RNA. Samples were sequenced on the Illumina Novaseq 6000. FastQC was used to determine the quality of the fastq files^70^. Transcript alignment/quantification was performed with Kallisto using default settings^71^. Ensembl annotation v86 was used as the reference transcriptome. Differential expression analysis was performed using Deseq2 with default settings^72^. Enrichment analysis was performed using the fgsea R package^73^. fgsea is based on the original GSEA method^74^. Genes were ranked using the log2 fold change (log2FC). Only genes with an absolute log2FC of >0.5 and adjusted *p*-value of <0.01 were used. Benjamini-Hochberg was used to compute the adjusted p-values. The Tsoi 2018 undifferentiated gene set included 224 genes belonging to “undifferentiated” and “undifferentiated-neural crest like” listed in Table S3 of that study^16^. The Venn diagram in Fig. 4c was made using the ggvenn R package^75^.

### CRISPR dependency scores

Depmap webtool (https://depmap.org/portal/) was used to generate Supplementary Fig. 7.

### Derivation of small gene set

We selected TEADs and MITF target genes with confirmed binding of these transcription factors in melanomas, which decreased upon knockdown of these transcription factors. We derived these genes from two studies on the regulatory landscape of cutaneous melanoma^31,37^. The gene expression values were converted into binary by setting those samples with the top tertile of expression to one and the rest to zero.

### Naive Bayes classifier

We used the e1071 R package to implement the Naive Bayes classifier with Laplace smoothing^76^. The training data consisted of 472 SKCM TCGA samples that were labeled as de-differentiated or differentiated based on k-means clustering of ~400 invasive and proliferative genes previously described. The test data consisted of 49 cutaneous melanoma cell line samples from CCLE. Again, the correct labels were determined by k-means clustering of ~400 invasive and proliferative genes previously described.

### Graphics

Figure 5a was created using BioRender.com.

### Reporting summary

Further information on research design is available in the Nature Research Reporting Summary linked to this article.

## Supplementary information

REPORTING SUMMARY

Supplementary figures

Supplementary data

## Data Availability

Untreated cell line gene expression and BRAFi (PLX4720) sensitivity data (AUC scores) were obtained from CCLE^77^. Gene expression values were extracted from “CCLE_RNAseq_genes_counts_20180929.gct.gz” and BRAFi sensitivity, i.e., the AUC for PLX4720, was extracted from “CCLE_NP24.2009_Drug_data_2015.02.24.csv.” RPPA protein values were extracted from “CCLE_RPPA_20180123.csv”. Patient BRAFi response and gene expression data were obtained from GSE77940^34^. Cell line ICI-resistance scores were obtained from GSE115978^50^. Patient PD-1 response and gene expression data were obtained from GSE65186^51^. TCGA patient data were extracted from the TCGA SKCM dataset using the GDCquery R package. The settings for GDCquery were project = ”TCGA-SKCM”, data.category = “Transcriptome Profiling”, data.type = “Gene Expression Quantification”, and workflow.type = “HTSeq - Counts”. Raw counts were normalized using the DESeqDataSetFromMatrix function with default settings. MITF and TEADs ChIP- and RNA-seq data were obtained from Supplementary Data of Verfaillie et al.^24^. Raw RNAseq files for the SRCi and *RAC1*-KD experiment can be obtained from the Sequence Read Archive, using the unique identifier: PRJNA861997.

## Code availability

Bioinformatic analysis was performed with custom R scripts using R version 3.6.0. These scripts can be obtained from github or by request from the authors.

## References

[1] Dummer R (2018). Encorafenib plus binimetinib versus vemurafenib or encorafenib in patients with BRAF-mutant melanoma (COLUMBUS): a multicentre, open-label, randomised phase 3 trial. Lancet Oncol..

[2] Marei H, Malliri A (2017). Rac1 in human diseases: the therapeutic potential of targeting Rac1 signaling regulatory mechanisms. Small GTPases.

[3] Davis MJ (2013). RAC1P29S is a spontaneously activating cancer-associated GTPase. Proc. Natl Acad. Sci. USA.

[4] Kawazu M (2013). Transforming mutations of RAC guanosine triphosphatases in human cancers. Proc. Natl Acad. Sci. USA.

[5] Watson IR (2014). The RAC1 P29S hotspot mutation in melanoma confers resistance to pharmacological inhibition of RAF. Cancer Res..

[6] Mohan AS (2019). Enhanced dendritic actin network formation in extended lamellipodia drives proliferation in growth-challenged Rac1(P29S) melanoma cells. Dev. Cell.

[7] Cerami E (2012). The cBio cancer genomics portal: an open platform for exploring multidimensional cancer genomics data. Cancer Discov..

[8] Gao J (2013). Integrative analysis of complex cancer genomics and clinical profiles using the cBioPortal. Sci. Signal.

[9] Feddersen CR (2019). Src-dependent DBL family members drive resistance to vemurafenib in human melanoma. Cancer Res..

[10] Vanneste M (2020). Functional genomic screening independently identifies CUL3 as a mediator of vemurafenib resistance via Src-Rac1 signaling axis. Front. Oncol..

[11] Zhu EY, Dupuy AJ (2022). Machine learning approach informs biology of cancer drug response. BMC Bioinformatics.

[12] Lu H (2017). PAK signalling drives acquired drug resistance to MAPK inhibitors in BRAF-mutant melanomas. Nature.

[13] Lionarons DA (2019). RAC1(P29S) induces a mesenchymal phenotypic switch via serum response factor to promote melanoma development and therapy resistance. Cancer Cell.

[14] Misek SA (2020). Rho-mediated signaling promotes BRAF inhibitor resistance in de-differentiated melanoma cells. Oncogene.

[15] Hoek KS (2006). Metastatic potential of melanomas defined by specific gene expression profiles with no BRAF signature. Pigment Cell Res..

[16] Tsoi J (2018). Multi-stage differentiation defines melanoma subtypes with differential vulnerability to drug-induced iron-dependent oxidative stress. Cancer Cell.

[17] Fallahi-Sichani M (2015). Systematic analysis of BRAF(V600E) melanomas reveals a role for JNK/c-Jun pathway in adaptive resistance to drug-induced apoptosis. Mol. Syst. Biol..

[18] Richard G (2016). ZEB1-mediated melanoma cell plasticity enhances resistance to MAPK inhibitors. EMBO Mol. Med..

[19] Sun C (2014). Reversible and adaptive resistance to BRAF(V600E) inhibition in melanoma. Nature.

[20] Titz B (2016). JUN dependency in distinct early and late BRAF inhibition adaptation states of melanoma. Cell Discov..

[21] Kim MH (2016). Actin remodeling confers BRAF inhibitor resistance to melanoma cells through YAP/TAZ activation. EMBO J..

[22] Lee JH (2020). Transcriptional downregulation of MHC class I and melanoma de- differentiation in resistance to PD-1 inhibition. Nat. Commun..

[23] Perez-Guijarro E (2020). Multimodel preclinical platform predicts clinical response of melanoma to immunotherapy. Nat. Med..

[24] Verfaillie A (2015). Decoding the regulatory landscape of melanoma reveals TEADS as regulators of the invasive cell state. Nat. Commun..

[25] Widmer DS (2012). Systematic classification of melanoma cells by phenotype-specific gene expression mapping. Pigment Cell Melanoma Res..

[26] Toyama Y, Kontani K, Katada T, Shimada I (2019). Decreased conformational stability in the oncogenic N92I mutant of Ras-related C3 botulinum toxin substrate 1. Sci. Adv..

[27] Johnson DB (2018). BRAF internal deletions and resistance to BRAF/MEK inhibitor therapy. Pigment Cell Melanoma Res..

[28] Park ER, Eblen ST, Catling AD (2007). MEK1 activation by PAK: a novel mechanism. Cell Signal.

[29] Slack-Davis JK (2003). PAK1 phosphorylation of MEK1 regulates fibronectin-stimulated MAPK activation. J. Cell Biol..

[30] Hugo W (2015). Non-genomic and immune evolution of melanoma acquiring MAPKi resistance. Cell.

[31] Song C (2017). Recurrent tumor cell-intrinsic and -extrinsic alterations during MAPKi-induced melanoma regression and early adaptation. Cancer Discov..

[32] Fallahi-Sichani M (2017). Adaptive resistance of melanoma cells to RAF inhibition via reversible induction of a slowly dividing de-differentiated state. Mol. Syst. Biol..

[33] Su Y (2017). Single-cell analysis resolves the cell state transition and signaling dynamics associated with melanoma drug-induced resistance. Proc. Natl Acad. Sci. USA.

[34] Tirosh I (2016). Dissecting the multicellular ecosystem of metastatic melanoma by single-cell RNA-seq. Science.

[35] Muller J (2014). Low MITF/AXL ratio predicts early resistance to multiple targeted drugs in melanoma. Nat. Commun..

[36] Marin-Bejar O (2021). Evolutionary predictability of genetic versus nongenetic resistance to anticancer drugs in melanoma. Cancer Cell.

[37] Anastas JN (2014). WNT5A enhances resistance of melanoma cells to targeted BRAF inhibitors. J. Clin. Invest..

[38] Konieczkowski DJ (2014). A melanoma cell state distinction influences sensitivity to MAPK pathway inhibitors. Cancer Discov..

[39] Shaffer SM (2017). Rare cell variability and drug-induced reprogramming as a mode of cancer drug resistance. Nature.

[40] Dilshat R (2021). MITF reprograms the extracellular matrix and focal adhesion in melanoma. Elife.

[41] Rausch V (2019). The hippo pathway regulates caveolae expression and mediates flow response via caveolae. Curr. Biol..

[42] Gray JL, von Delft F, Brennan PE (2020). Targeting the small GTPase superfamily through their regulatory proteins. Angew. Chem. Int. Ed..

[43] Girotti MR (2013). Inhibiting EGF receptor or SRC family kinase signaling overcomes BRAF inhibitor resistance in melanoma. Cancer Discov..

[44] Atefi M (2011). Reversing melanoma cross-resistance to BRAF and MEK inhibitors by co-targeting the AKT/mTOR pathway. PLoS ONE.

[45] Perna D (2015). BRAF inhibitor resistance mediated by the AKT pathway in an oncogenic BRAF mouse melanoma model. Proc. Natl Acad. Sci. USA.

[46] Ramsdale R (2015). The transcription cofactor c-JUN mediates phenotype switching and BRAF inhibitor resistance in melanoma. Sci. Signal.

[47] Hartman ML, Czyz M (2015). MITF in melanoma: mechanisms behind its expression and activity. Cell Mol. Life Sci..

[48] Rambow F (2015). New functional signatures for understanding melanoma biology from tumor cell lineage-specific analysis. Cell Rep..

[49] Gerami P (2015). Development of a prognostic genetic signature to predict the metastatic risk associated with cutaneous melanoma. Clin. Cancer Res..

[50] Jerby-Arnon L (2018). A cancer cell program promotes T cell exclusion and resistance to checkpoint blockade. Cell.

[51] Hugo W (2016). Genomic and transcriptomic features of response to anti-PD-1 therapy in metastatic melanoma. Cell.

[52] Nazarian R (2010). Melanomas acquire resistance to B-RAF(V600E) inhibition by RTK or N-RAS upregulation. Nature.

[53] Munoz-Alonso MJ (2008). Plitidepsin has a dual effect inhibiting cell cycle and inducing apoptosis via Rac1/c-Jun NH2-terminal kinase activation in human melanoma cells. J. Pharm. Exp. Ther..

[54] Shi H, Zhang CJ, Chen GY, Yao SQ (2012). Cell-based proteome profiling of potential dasatinib targets by use of affinity-based probes. J. Am. Chem. Soc..

[55] Hirata E (2015). Intravital imaging reveals how BRAF inhibition generates drug-tolerant microenvironments with high integrin beta1/FAK signaling. Cancer Cell.

[56] DerMardirossian C, Rocklin G, Seo JY, Bokoch GM (2006). Phosphorylation of RhoGDI by Src regulates Rho GTPase binding and cytosol-membrane cycling. Mol. Biol. Cell.

[57] Girard CA (2020). A feed-forward mechanosignaling loop confers resistance to therapies targeting the MAPK pathway in BRAF-mutant melanoma. Cancer Res..

[58] Lamar JM (2019). SRC tyrosine kinase activates the YAP/TAZ axis and thereby drives tumor growth and metastasis. J. Biol. Chem..

[59] Lin L (2015). The Hippo effector YAP promotes resistance to RAF- and MEK-targeted cancer therapies. Nat. Genet..

[60] Pham TH (2021). Machine-learning and chemicogenomics approach defines and predicts cross-talk of Hippo and MAPK pathways. Cancer Discov..

[61] Pearson JD (2021). Binary pan-cancer classes with distinct vulnerabilities defined by pro- or anti-cancer YAP/TEAD activity. Cancer Cell.

[62] Gibault F, Sturbaut M, Bailly F, Melnyk P, Cotelle P (2018). Targeting transcriptional enhanced associate domains (TEADs). J. Med. Chem..

[63] Khaliq M, Manikkam M, Martinez ED, Fallahi-Sichani M (2021). Epigenetic modulation reveals differentiation state specificity of oncogene addiction. Nat. Commun..

[64] Rambow F, Marine JC, Goding CR (2019). Melanoma plasticity and phenotypic diversity: therapeutic barriers and opportunities. Genes Dev..

[65] Belote RL (2021). Human melanocyte development and melanoma dedifferentiation at single-cell resolution. Nat. Cell Biol..

[66] Riesenberg S (2015). MITF and c-Jun antagonism interconnects melanoma dedifferentiation with pro-inflammatory cytokine responsiveness and myeloid cell recruitment. Nat. Commun..

[67] Boshuizen J (2020). Reversal of pre-existing NGFR-driven tumor and immune therapy resistance. Nat. Commun..

[68] Vu HL, Rosenbaum S, Purwin TJ, Davies MA, Aplin AE (2015). RAC1 P29S regulates PD-L1 expression in melanoma. Pigment Cell Melanoma Res..

[69] Wilson MH, Coates CJ, George AL (2007). PiggyBac transposon-mediated gene transfer in human cells. Mol. Ther..

[70] Andrews, S. FastQC: a quality control tool for high throughput sequence data. http://www.bioinformatics.babraham.ac.uk/projects/fastqc (2010).

[71] Bray NL, Pimentel H, Melsted P, Pachter L (2016). Near-optimal probabilistic RNA-seq quantification. Nat. Biotechnol..

[72] Love MI, Huber W, Anders S (2014). Moderated estimation of fold change and dispersion for RNA-seq data with DESeq2. Genome Biol..

[73] Korotkevich, G., Sukhov, V., Budin, N., Shpak, B., Artyomov, M. N. & Sergushichev, A. Fast gene set enrichment analysis. bioRxiv 060012 (2021).

[74] Subramanian A (2005). Gene set enrichment analysis: a knowledge-based approach for interpreting genome-wide expression profiles. Proc. Natl Acad. Sci. USA.

[75] Yan, L. An easy-to-use way to draw pretty venn diagram by ‘ggplot2’. https://cran.r-project.org/web/packages/ggvenn/index.html (2021).

[76] Meyer, D. et al. E1071: Misc Functions of the Department of Statistics (E1071). https://cran.r-project.org/web/packages/e1071/index.html (2009).

[77] Barretina J (2012). The Cancer Cell Line Encyclopedia enables predictive modelling of anticancer drug sensitivity. Nature.

