# Supplementary material for: SRC-RAC1 signaling drives drug resistance to BRAF inhibition in de-differentiated cutaneous melanomas
Source: NPJ Precis Oncol. 2022 Oct 21;6:74. doi: 10.1038/s41698-022-00310-7 (PMC9587254; doi:10.1038/s41698-022-00310-7)
Supplement: Supplementary file 2 — Supplementary figures [file 41698_2022_310_MOESM2_ESM.pdf]

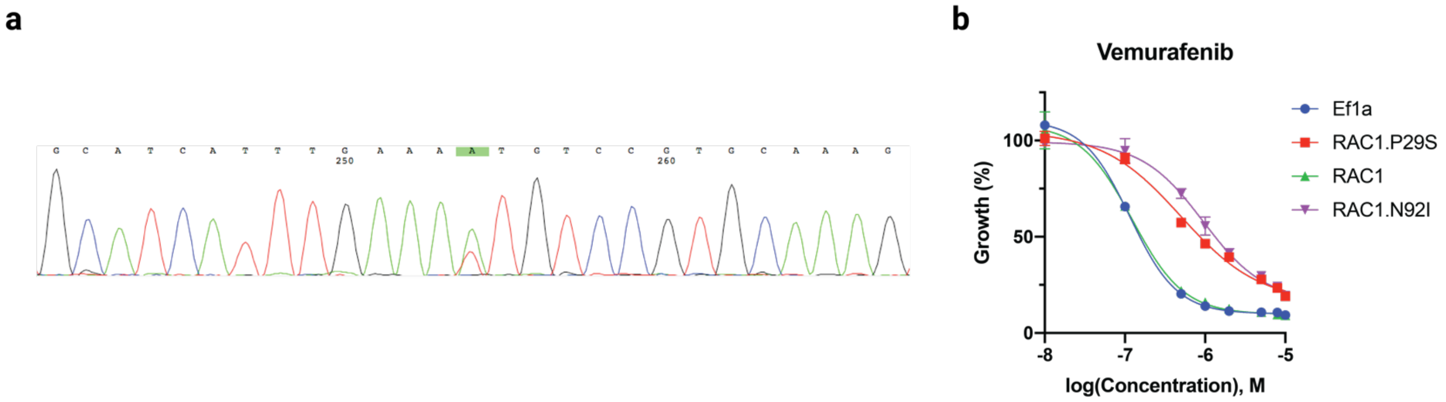

Supplementary Figure 1. **Sanger sequencing of VRPP3 and functional assessment of *RAC1*^{N92I} mutation.** (**a**) Sanger sequencing analysis of RAC1 in VRPP3. (**b**) VEM dose-response curve of A375 with enforced expression of empty vector, *RAC1, RAC1*^{P29S}, or *RAC1*^{N92I}. Ef1a denotes the empty vector and stands for the EF1-alpha promoter. Error bars in this figure denote the standard deviation.

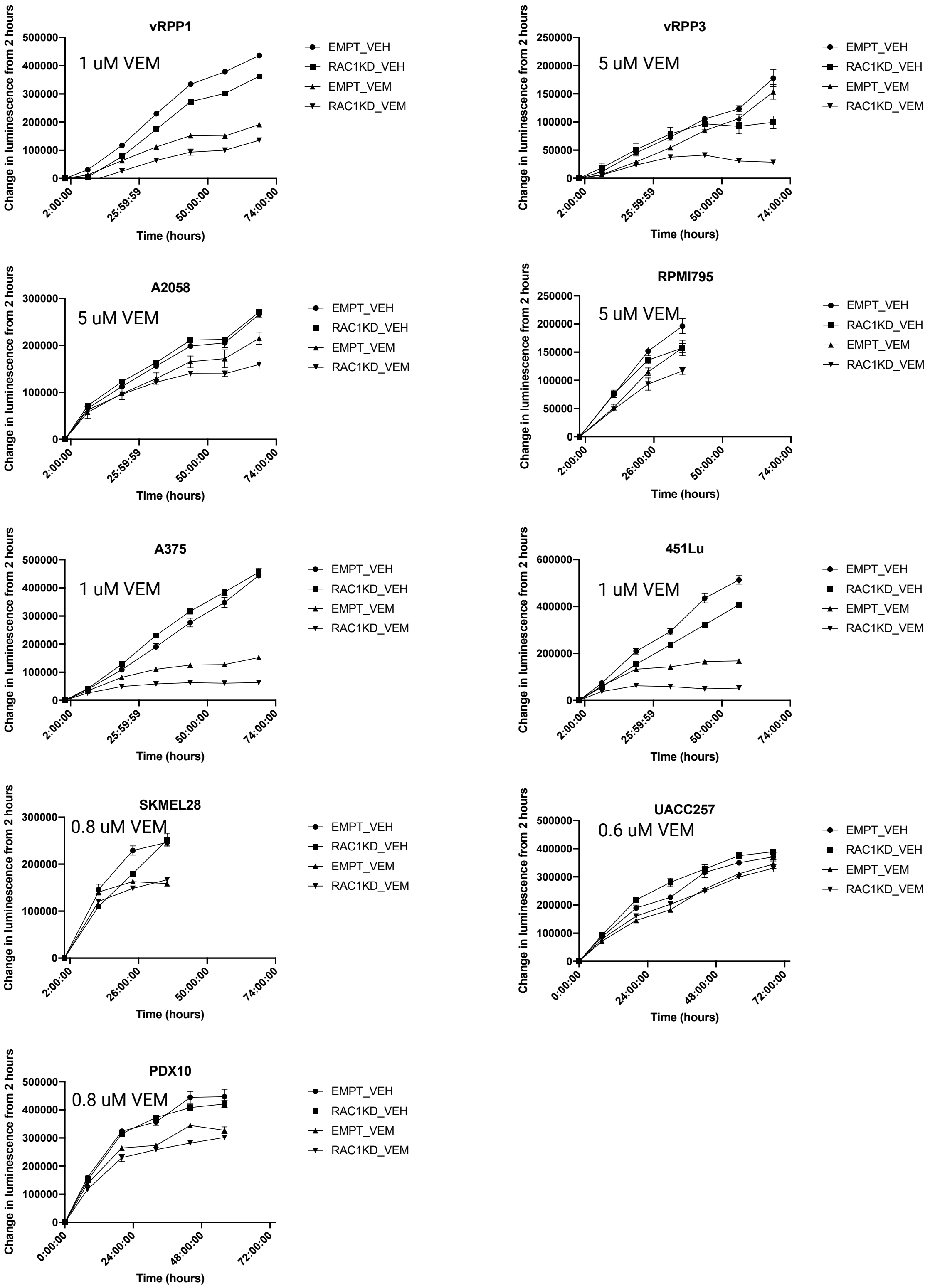

Supplementary Figure 2. Real-time viability of cell lines with transduced with *RAC1*-targeting versus none-targeting shRNAs. Y axis represents the difference of the luminescence at the indicated timepoint with respect to a two-hour reading. Data shown up until maximum luminescence reading. Last reading took place at 72 hours. Cell lines are differentially sensitive to VEM, so different concentrations were used to better show the effect of *RAC1*-knockdown. The concentration of VEM used for each cell line is indicated in the graph. Error bars in this figure denote the standard deviation.

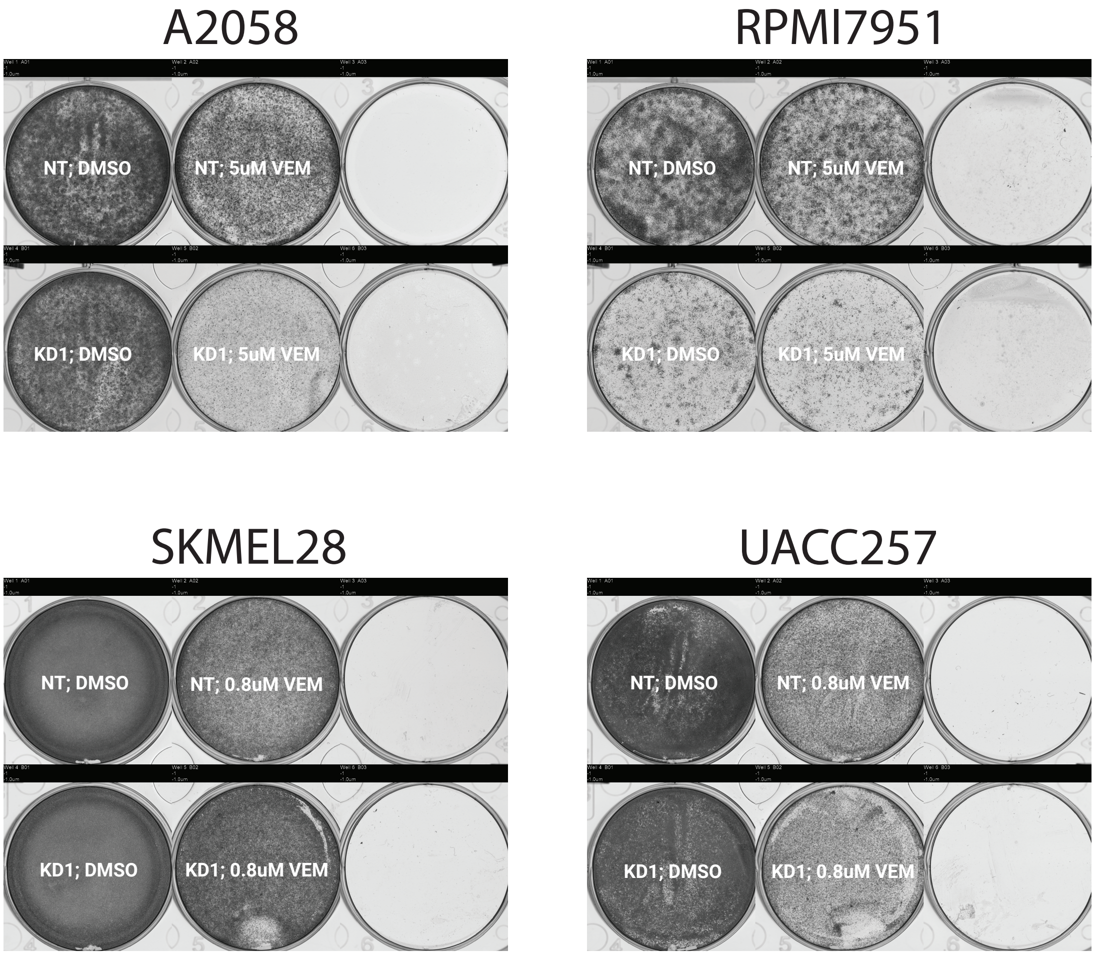

Supplementary Figure 3. **Growth of cell lines with transduced with *RAC1*-targeting versus none-targeting shRNAs in a six-well format.** Each well has the indicated shRNA that was transduced and the drug treatment condition. In contrast to the 96-well experiment, cells shown here were exposed to VEM or DMSO for a period of 5 days instead of 3 days (NT = non-targeting shRNA, KD1= *RAC1*-targeting shRNA).

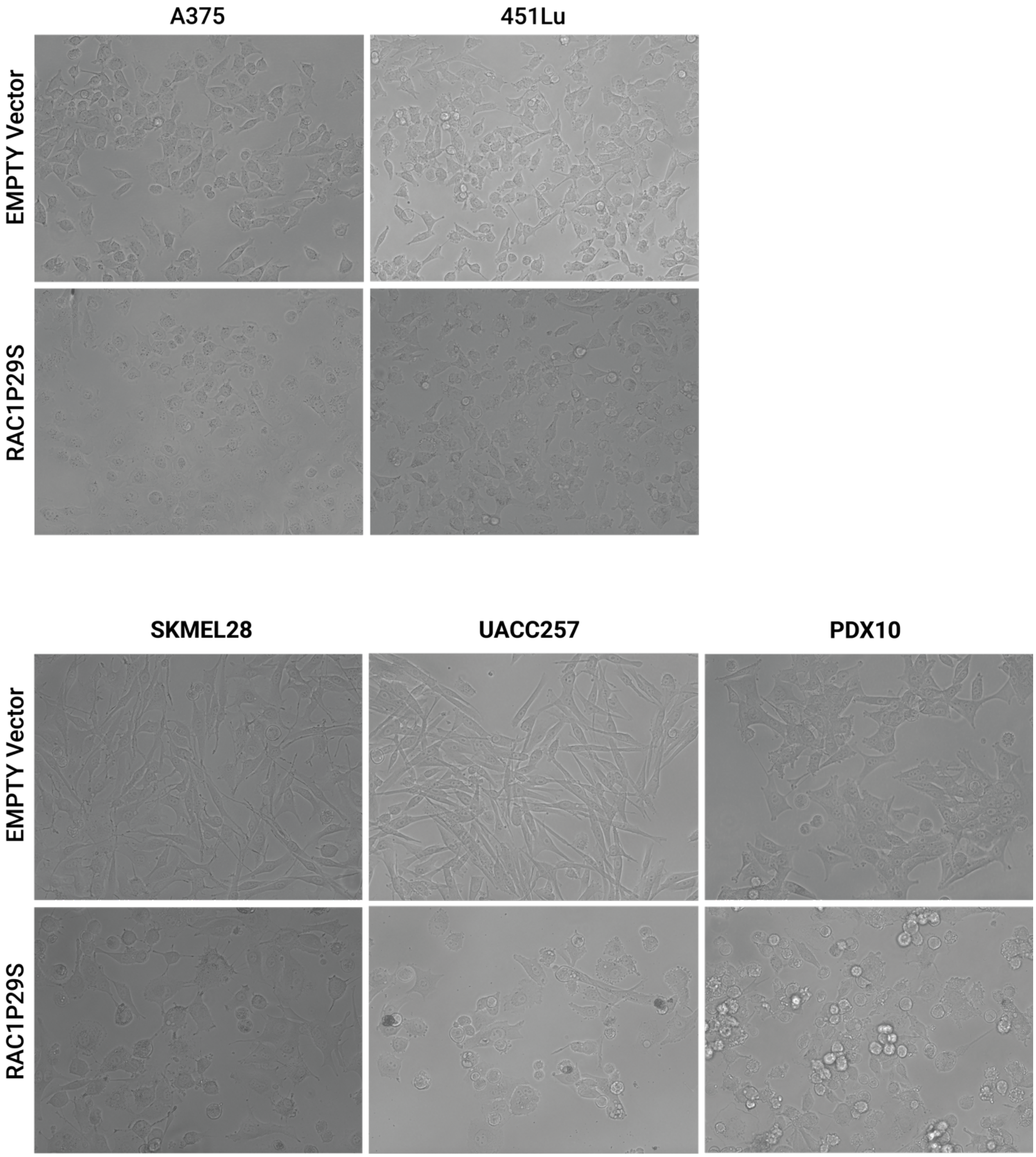

Supplementary Figure 4. **Images of cell lines with overexpression of *RAC1*^{P29S} or empty vector.**

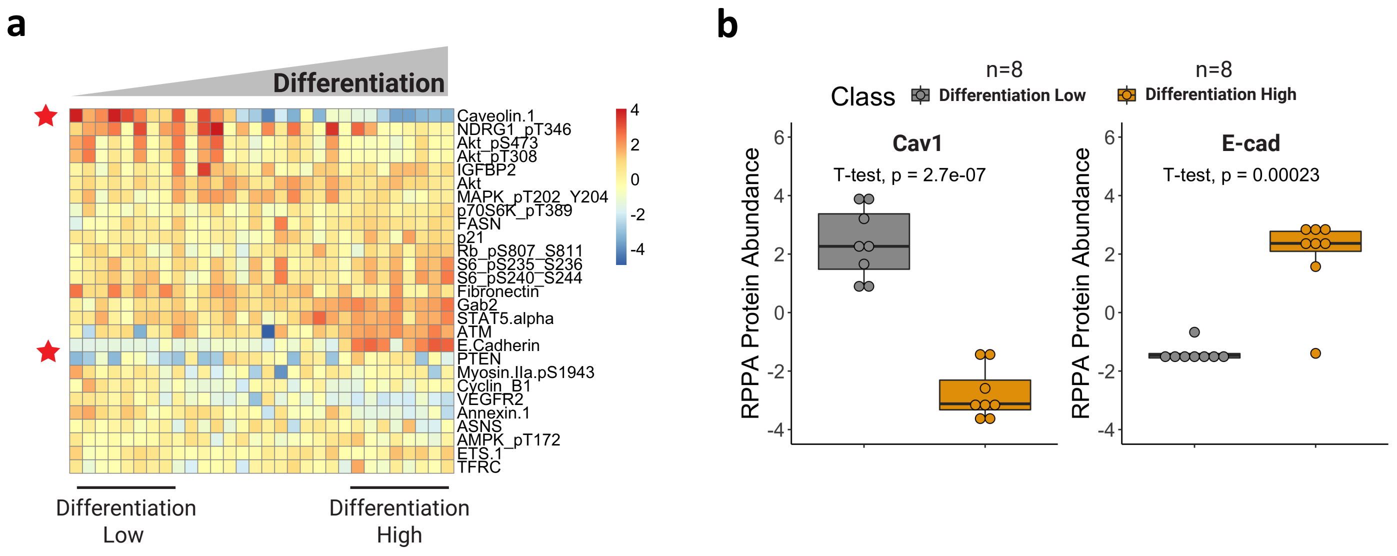

Supplementary Figure 5. **Importance of Cav1 and E-cadherin for response to BRAFi.** (**a**) Normalized RPPA protein abundance of top variably expressed protein across *BRAF*^{V600} melanomas. Red star highlights most differentially expressed proteins Cav1 and Ecad. (**b**) Comparison of Cav1 and E-cadherin protein abundance between the eight low- and eight high- differentiation. Alternative visualization of data presented in (**a**). Error bars in this figure denote the standard deviation. Asterisk denotes unpaired two-tailed student's t-test p-value of < 0.05.

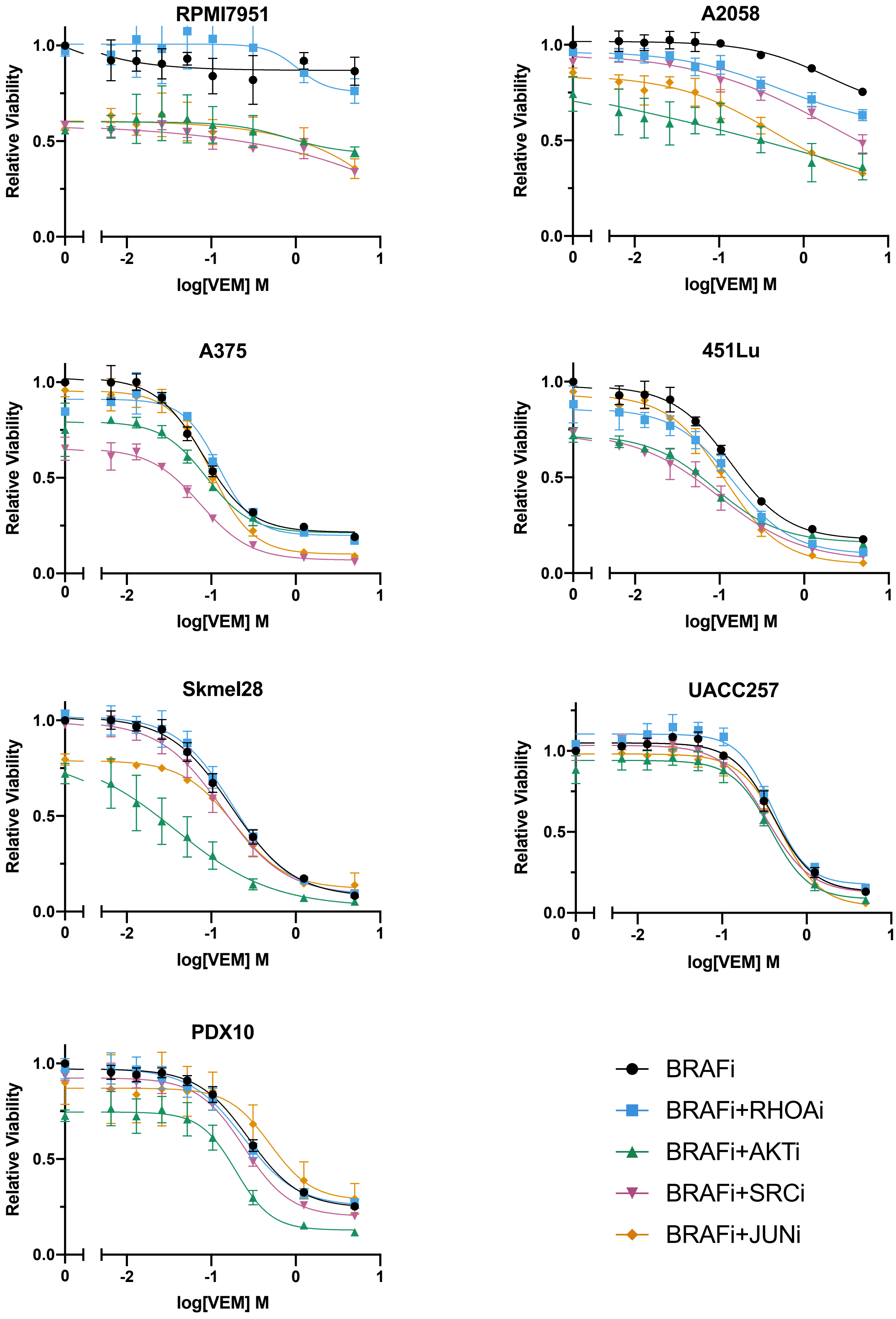

Supplementary Figure 6. Dose-response curves for indicated drug combinations for a give melanoma cell line. Viability is normalized to vehicle treated cells. Error bars in this figure denote the standard deviation.

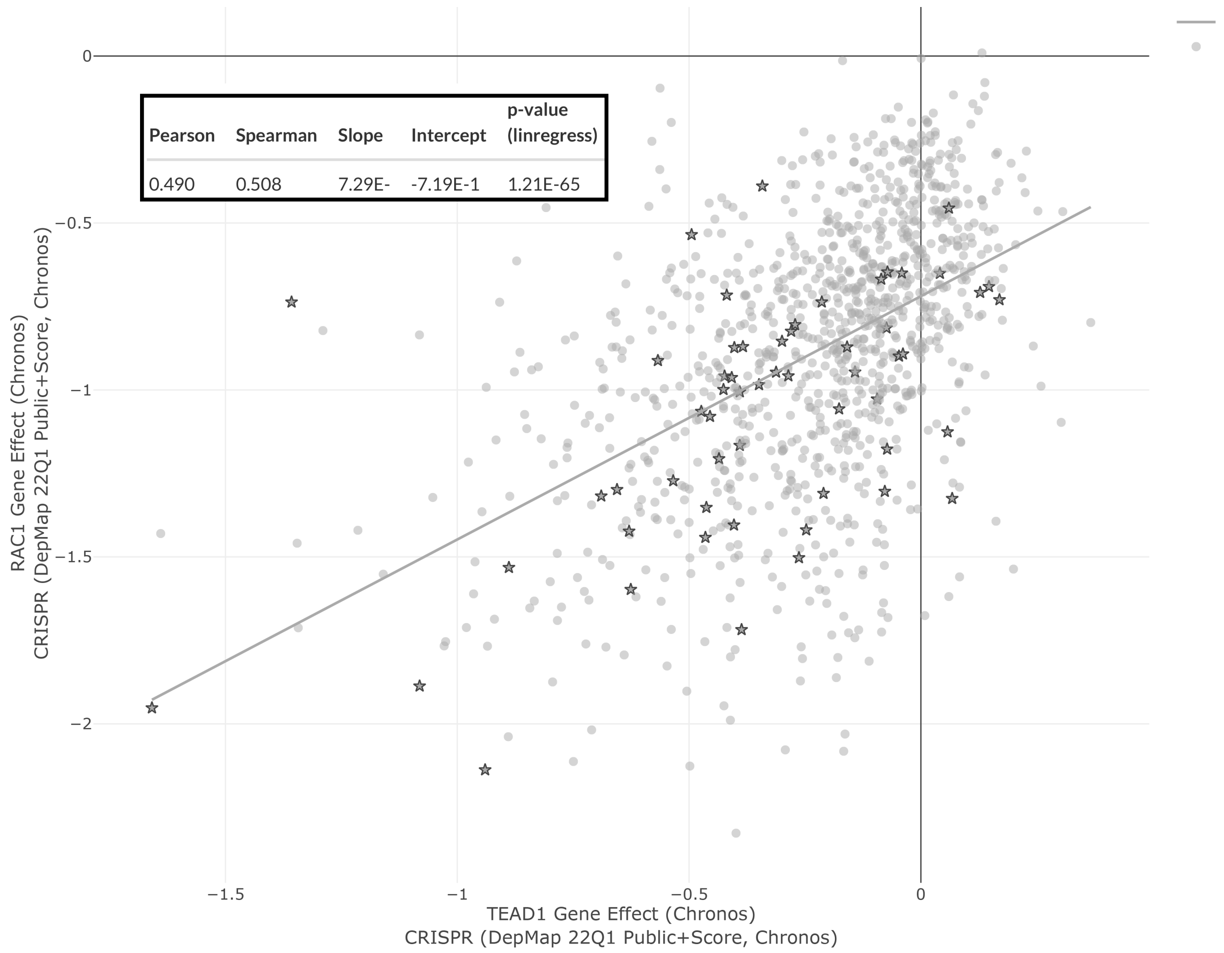

Supplementary Figure 7. Association between RAC1- and TEAD1- dependency across cancers. CRIPSR dependency scores for RAC1 and TEAD1 is given by the y- and x- axis respectively. The stars indicate cutaneous melanoma cell lines. Dependency increases as scores decrease.

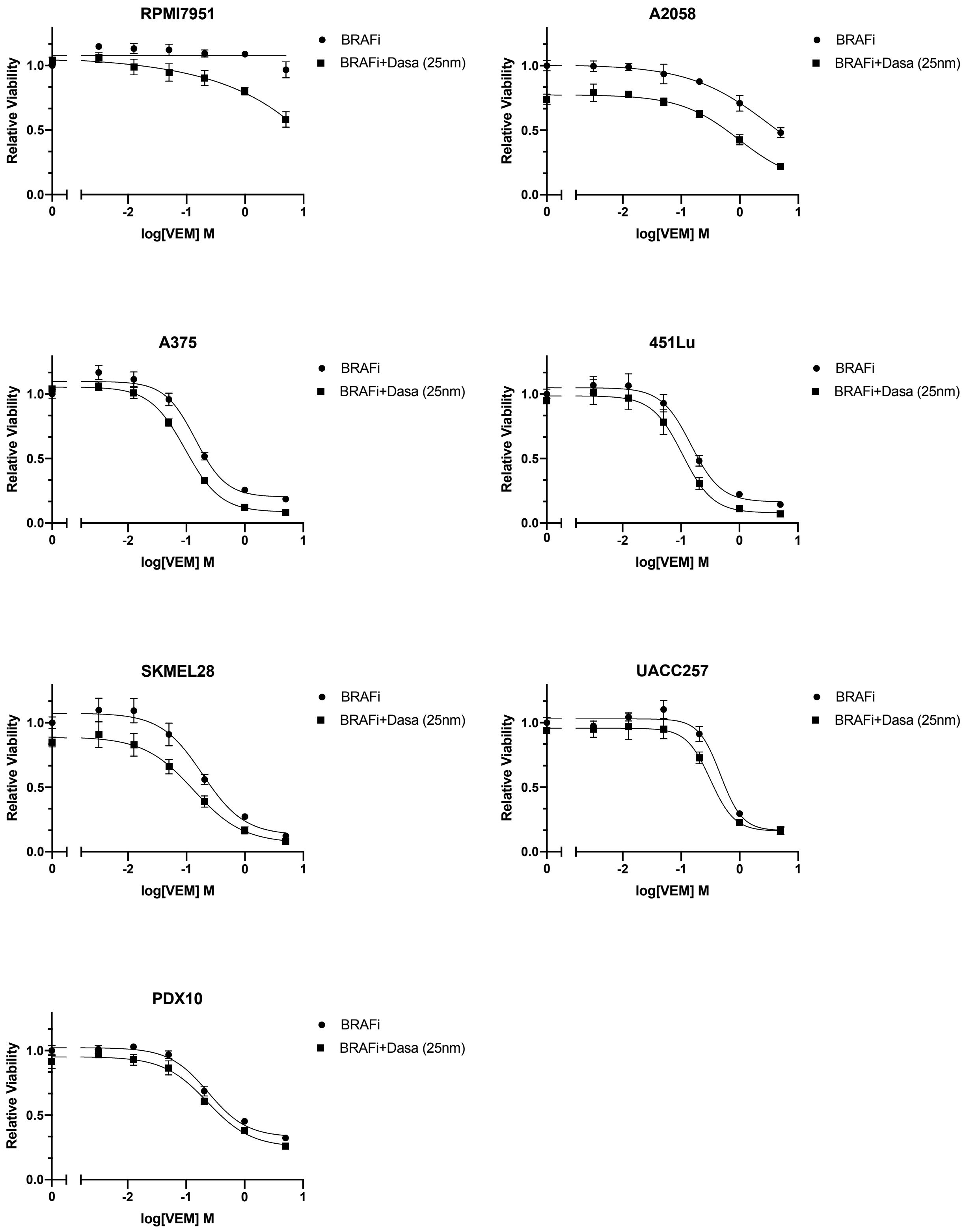

Supplementary Figure 8. Efficacy of VEM and dasatinib combination. Viability is normalized to vehicle treated cells. Error bars in this figure denote the standard deviation.

Fig. 1a B-actin 45 kDa; Tub, 50 kDa; Rac1 20 kDa

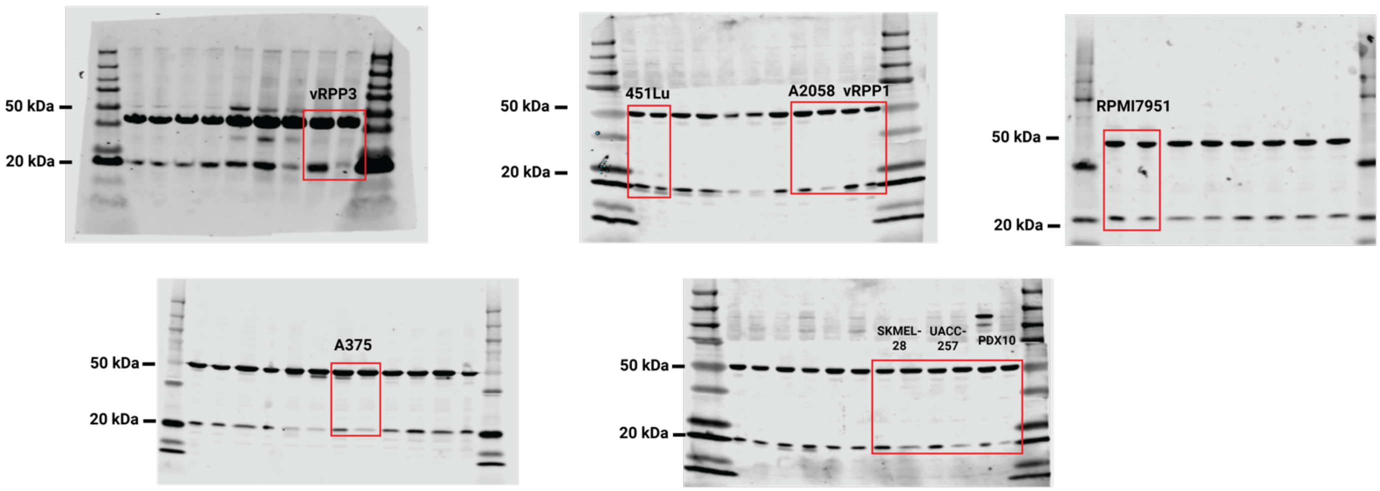

Fig. 1d Tub, 50 kDa; Rac1 20 kDa

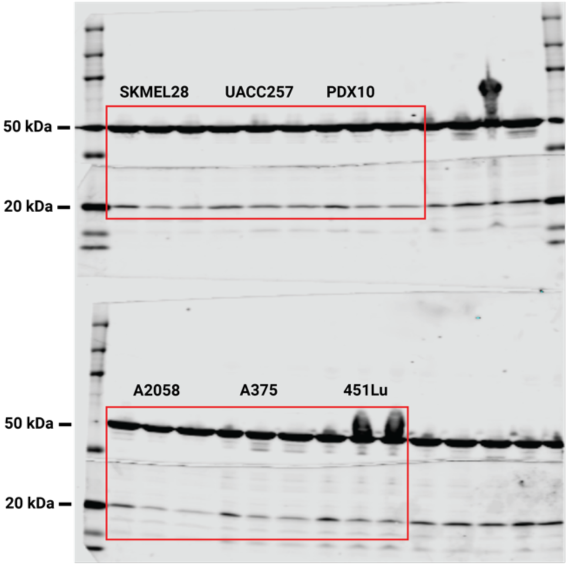

Fig. 1f Tub, 50 kDa; Rac1 20 kDa

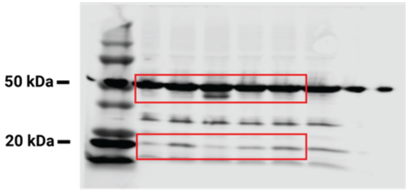

Fig.2a Tub, 50 kDa; Rac1 20 kDa, MEK 45 kDa

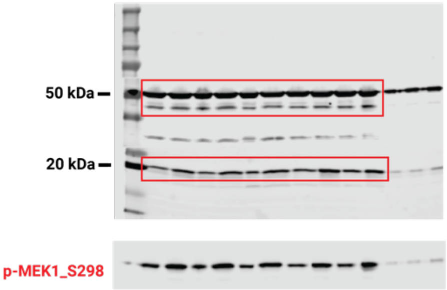

Fig. 3b Ecad, 130 kDA; Axl 138 kDA; Tub, 50 kDa; Rac1 20 kDa; Cav1 20 kDa

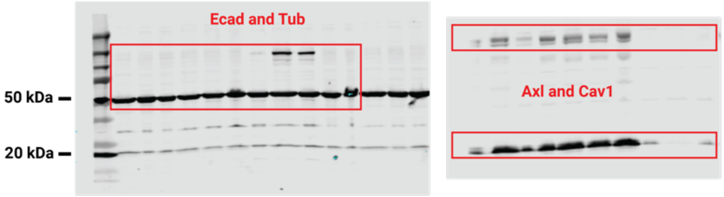

Fig.3c Axl, 138 kDa; Tub, 50 kDa; Rac1 20 kDa; Cav1 20 kDa

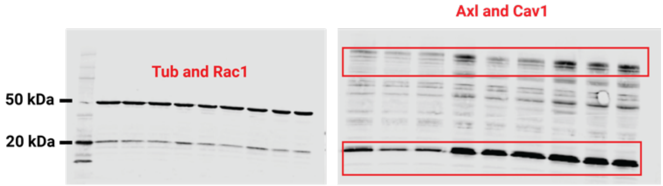

Fig.3d Axl, 138 kDa; Tub, 50 kDa; Rac1 20 kDa, Cav1 20 kDa

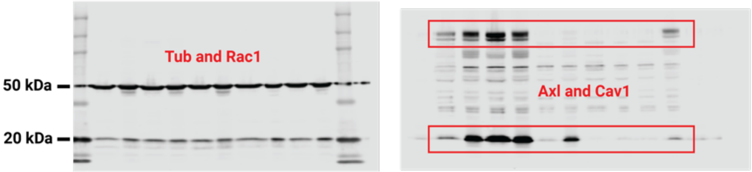

Supplementary Figure 9. Uncropped blots.
